# Comparison of Contrast-Enhanced Isotropic 3D-GRE-T1WI Sequence versus Conventional Non-Isotropic Sequence on Preoperative Staging of Cervical Cancer

**DOI:** 10.1371/journal.pone.0122053

**Published:** 2015-03-23

**Authors:** Xiaoduo Yu, Meng Lin, Feng Ye, Han Ouyang, Yan Chen, Chunwu Zhou, Zihua Su

**Affiliations:** 1 Department of Diagnostic Radiology, Cancer Institute & Hospital, Peking Union Medical College, Chinese Academy of Medical Sciences, Beijing, China; 2 Life Science, GE Healthcare, Beijing, China; ACTREC (Advanced Centre for Treatment, Research and Education in Cancer) / Tata Memorial Centre, INDIA

## Abstract

**Purpose:**

To compare contrast-enhanced isotropic 3D-GRE-T1WI sequence vs. conventional non-isotropic sequence in terms of image quality, estimated signal-to-noise ratio (eSNR), relative tumor contrast and performance of cervical cancer staging.

**Methods:**

This retrospective study was approved by the institutional review board, and informed consent was waived. Seventy-one patients (47 ± 9.4 years), with pathologically-confirmed cervical cancer underwent axial contrast-enhanced 1mm^3^ isotropic 3D-GRE-T1WI sequence (herein referred to Isotropy), and 3-mm-thick non-isotropic sagittal and coronal sequences. Image quality score, eSNR and relative contrast between tumor to myometrium, gluteal muscle, and fat respectively, were compared between 3-mm-thick reconstructed images from Isotropy and directly scanned non-isotropic images by paired t-test. Difference in tumor staging obtained from Isotropy and combined Three-planes including reconstructed axial images, directly scanned sagittal and coronal sequence were compared by McNemar test.

**Results:**

Both sequences showed similar image quality. Reconstructed images demonstrated higher eSNR, equal or lower relative tumor contrast compared with non-isotropic images. Compared with performing diagnosis on Three-planes, both reviewers showed higher accuracy when diagnosing vaginal invasion on Isotropy (p = 0.039 and 0.003, respectively).

**Conclusion:**

Compared with non-isotropic sequence, 3.0T MR isotropic 3D-GRE-T1WI sequence exhibited better eSNR, providing more reliable clinical information for preoperative staging of cervical cancer.

## Introduction

Cervical cancer is the third most common gynecologic malignancy. Cervical cancer treatment includes surgery, radiotherapy and chemotherapy. Pretreatment tumor stage is vital to treatment decision and prognosis.

Magnetic resonance imaging (MRI) provides excellent soft tissue resolution and is able to delineate the normal zonal anatomy of cervix, local extent of cervical tumor and regional lymph node metastasis. MRI has been widely considered to be optimal imaging method for cervical cancer as an important adjunct to help clinical treatment planning [1–3]. With the development of new MRI technique, MRI has enhanced its role in cervical tumor imaging. One of these promising techniques is three-dimensional (3D) isotropic sequence. This technique has been widely applied on brain, vertebrae, joints, abdomen, and pelvis, where 3D isotropic sequence demonstrated better image quality compared with traditional two-dimensional (2D) sequences [4–10]. In addition, 3D isotropic high-resolution scan can provide MPR images at any angle, which not only provides more detailed anatomical structure, and better performance in lesion detection, diagnosis and staging, but also simplified scanning process.

T2 weighted imaging (T2WI) and contrast-enhanced T1WI are important sequences for staging of cervical cancer, with a diagnostic sensitivity of approximately 75% to 92% [11–14] and 95% [15] respectively. Furthermore, Akita et al. [15] showed that contrast-enhanced T1WI showed higher contrast to noise ratio (CNR) than T2WI, which allowed easier detection of the small cervical cancer lesions. However, a comprehensive study on the diagnostic value of 3D isotropic contrast-enhanced T1WI on cervical cancer has yet to be performed. The purpose of this study was to compare the 3.0T MR contrast-enhanced isotropic 3D gradient recalled echo T1 weighted imaging (3D-GRE-T1WI) sequence vs. non-isotropic sequence in terms of image quality, eSNR, relative tumor contrast, and staging of cervical cancer to assess the value of the 3D isotropic contrast-enhanced sequence in the preoperative staging of cervical cancer.

## Materials and Methods

### Patients

This retrospective study was approved by the institutional review board at Cancer Institute & Hospital, Peking Union Medical College, and Chinese Academy of Medical Sciences with waiver of informed consent. Since a couple of the patients’ images were shown in this paper, those patients have provided written informed consent for publication. All patient information was anonymized and de-identified prior to analysis. From April 2009 to September 2010, there were a total of 234 patients with newly diagnosed cervical cancer examined by MRI in our hospital. Eighty-nine cases underwent hysterectomy or surgical biopsy, where pathological stage results can be obtained. Ten cases only conducted lymph node dissection due to local advanced disease. Ninety-four cases underwent concurrent radiochemotherapy. Six cases were treated by chemotherapy. Thirty-five cases have been performed with unknown treatment in other hospitals.

Of the eighty-nine cases, which performed surgery in our hospital, eighteen patients were excluded: six patients had unenhanced MR scan, one underwent scanning on 1.5T MR device, six were conducted preoperative radiochemotherapy and five did not undergo 3D isotropic sequence. The remaining 71 patients were enrolled in this study (age range, 28–71 years; mean age: 47 ± 9.4 years), with cervical tumor (diameter range, 0.9–5.9cm; mean diameter, 2.97 ± 1.23 cm) on preoperative MRI.

Surgeries were performed on these 71 patients three to seven days after MR scans. Seventy patients underwent extensive hysterectomy and pelvic lymph node dissection and one patient had only lymph node dissection due to a positive biopsy finding of tumor between uterus and bladder.

Postoperative pathological findings included squamous cell carcinoma in 61 cases (86%), adenocarcinoma in 8 cases (11%), adenosquamous carcinoma in 2 cases (3%). According to the 2009 FIGO Staging Criteria [16], the postoperative pathological stages included 47 cases of stage I B1, 11 cases of stage I B2, 10 cases of stage II A (with involvement of the upper two-thirds of the vagina, without parametrial invasion), and three cases of stage II B with parametrial invasion. All three cases of stage II B had no vaginal invasion.

### MR protocol

All MR scans were conducted on a 3.0T MRI scanner (Signa excite HD, GE, USA) with an 8-channel phased array coil. All patients received intramuscular injection of 20 mg/ml scopolamine butylbromide at 10 minutes before MR scanning to prevent gastrointestinal motility. In addition, an OB tampons was utilized to make vagina dilated.

A series of unenhanced MRI sequence were performed first, which includes axial fast recovery fast spin echo (FRFSE) sequence T1WI, axial and sagittal FRFSE T2WI, axial pre-saturated fat suppression FRFSE T2WI sequence and a Diffusion-weighted imaging (DWI) sequence. Dynamic contrast-enhanced MRI (DCE-MRI) was then performed for 240 s by 3D-GRE-T1WI [liver acquisition with volume acceleration-extended volume (LAVA-XV)] after injection of Gadolinium-DTPA (Gd-DTPA) with dose of 0.2 ml/kg and rate of 2 ml/s. In the end, sagittal, axial and coronal series were performed (Table 1). It can be seen that the axial series was isotropic with 1 mm^3^ resolution and other two are non-isotropic with 3-mm-thick. Our comparisons were performed between these three series.

**Table 1:** Parameters of contrast-enhanced sagittal, axial isotropic and coronal scans.

Sequence	Scan direction	TR (ms)	TE (ms)	Bandwidth	Flip angle (°)	FOV (cm)	Matrix size	Thickness (mm)	Scan time (s)
Sagittal anisotropy	Along the long axis of the uterus	3.7	1.7	83.33	15	34	320×192	3	40
Axial Isotropy	Perpendicular to the body axis	4.2	1.9	83.33	15	35	350×350	1	150
Coronal anisotropy	Along the long axis of the uterus	3.7	1.7	83.33	15	35	320×192	3	35

Note: Scan slices varied according to patient’s body size, therefore, the scan time for each patient may have also varied. Sagittal and coronal scans covered the entire pelvic cavity and the axial scan covered the entire uterus and vagina.

### Data analysis

All data were processed by using Advantage Workstation (ADW 4.4 version, GE). The axial isotropic sequences were first reconstructed to 3-mm-thick in both sagittal and coronal plane; therefore, comparisons can be performed in corresponding views.

### Image quality

Two radiologists (H.O. and M.L.), with experience in body tumor MRI for 20 years (H.O.) and 11 years (M.L.), assessed image quality. Evaluation criteria is based on image uniformity and presence of artifacts with a five scale scores [1 = unacceptable (diagnosis cannot be made), 2 = poor (artifacts are apparent and image is blurred), 3 = fair (there are artifacts, but clinical observation is not affected), 4 = good (few artifacts), and 5 = excellent (no artifacts)].

### eSNR and relative tumor contrast

A third radiologist (F.Y.) with 8 years of experience in body tumor MRI, who was blinded to the sequence information, measured and calculated the eSNR and relative tumor contrast. Due to the unevenness of noise distribution in the FOV after using the parallel acquisition sequence, the noise component in SNR cannot be directly measured from air [17]. Accordingly, we calculated the eSNR using the formula, myometrial SI/standard deviation, which are obtained from myometrial ROI and SI stands for signal intensity.

Comparison between tumor and surrounding tissue signals was reflected by the relative tumor contrast [9] using the formula: |(A-B)|/ (A+B), where A and B were the absolute intensity values of the tumor and surrounding tissues.

Tumor ROI was placed on the entire tumor on the slice, where largest area of tumor presents. Myometrium ROI was placed on the normal myometrium adjacent to the tumor with areas of 0.13–1.83 cm^2^ (0.59±0.51 cm^2^), and both fibroids and adenomyosis were avoided. Gluteus ROI was placed on the gluteus maximus muscle, avoiding the fat, with areas of 0.18–1.91 cm^2^ (0.64±0.29 cm^2^). Fat ROI was placed on the fat tissue in the anterior (on sagittal plane) or lateral abdominal wall (on coronal plane) with areas of 0.19–1.33 cm^2^ (0.49±0.20 cm^2^).

### Local staging

Two radiologists (H.O. and M.L.) were invited to perform FIGO staging. Isotropic sequence [hereafter Isotropy, with MPR, curved planar reformation (CPR)] was firstly used for staging. Two months later, the same two radiologists performed staging with other sequence (hereafter Three-planes), which are 3-mm-thick axial images reconstructed from isotropic sequence, conventional 3-mm-thick directly scanned non-isotropic sagittal and coronal sequences. They were blinded to all patient information. Unenhanced series including T1WI, T2WI and DWI were also used during every assessment as complementary information. Vaginal invasion is defined as tumor extending to and interrupted the vaginal wall. Parametrial invasion is concluded if cervical stromal ring is disruptive and tumor protrudes into parametrium.

### Statistical analysis

All data were analyzed using SPSS13.0. Kolmogorov-Smirnov test was performed to examine the normality of the image quality score, the eSNR, and relative tumor contrast. From results, it was shown that image quality scores were not normally distributed, but other data complied with normal distribution. We used non-parametric Wilcoxon paired test for the image quality score, paired sample *t*-test for eSNR and relative tumor contrast to compare the non-isotropic sequence vs. isotropic sequence. McNemar test was used to compare the accuracy of the Isotropy vs. Three-planes when performing assessment of vaginal and parametrial invasion. Results were considered to be significant, when *p* value was < 0.05.

## Results

### Image quality score

The image quality scores for both sagittal and coronal contrast-enhanced directly acquired images vs. reconstructed images in same plane were not statistically different (Table 2).

**Table 2:** Image quality scores.

Scores	1	2	3	4	5	p	t
Directly acquired contrast-enhanced sagittal images	0	1(1)	6(8)	64(90)	0	0.180	-1.342
Reconstructed contrast-enhanced sagittal images	0	2(3)	7(10)	62(87)	0		
Directly acquired contrast-enhanced coronal images	0	2(3)	6(8)	63(89)	0	0.564	-0.577
Reconstructed contrast-enhanced coronal images	0	2(3)	7(10)	62(87)	0		

Note: The numbers in parentheses are the percentages of cases among the total number of cases.

### eSNR

**Fig 1:**
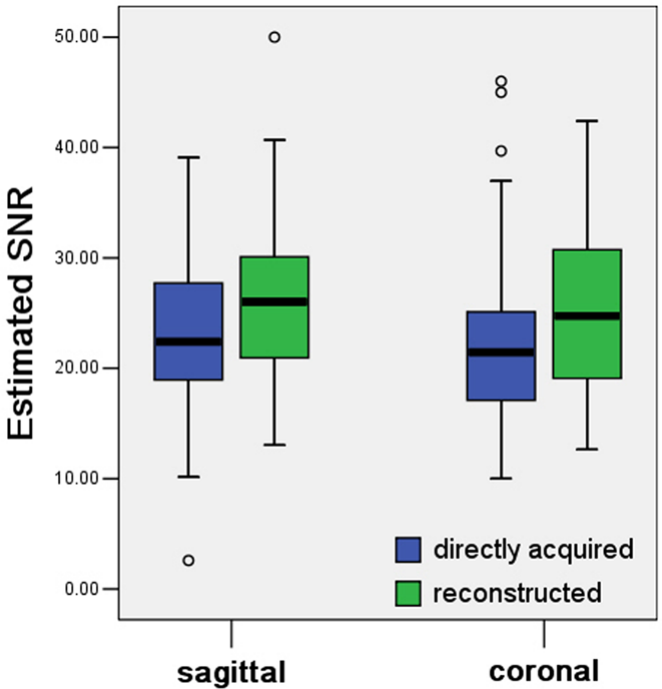
Box and whisker plots of estimated SNR of myometrium. The estimated SNRs of the myometrium in reconstructed images are higher than those in directly acquired images of both sagittal and coronal plane (*p* = 0.001 and 0.002, respectively).

**Fig 2:**
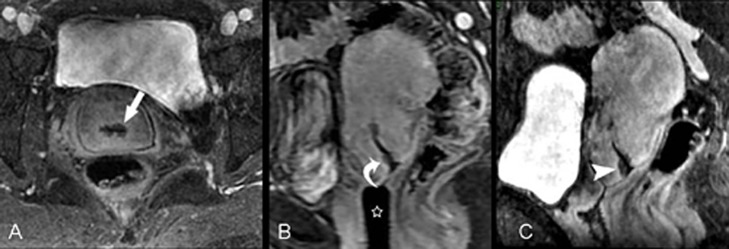
F48, poorly differentiated squamous cell carcinoma (stage IB1). Contrast-enhanced axial isotropic sequence (A) shows a low signal lesion around the cervical canal (arrow); the signal is lower than that of the surrounding cervical stroma and the greatest depth is approximately 4.4 mm. Directly acquired contrast-enhanced sagittal images (B) show a suspicious low signal lesion in the cervical canal close to the external os of the uterus (curved arrow). An OB tampons was placed inside the vagina (star). Oblique sagittal images reconstructed by the Isotropy (C) reveal the boundary of the cervical lesion (arrow head) more clearly, improving diagnostic confidence.

Contrast-enhanced reconstructed images had a higher eSNR compared to the contrast-enhanced directly acquired images in both the sagittal and coronal plane (Table 3, Fig. 1 and 2).

**Table 3:** Estimated SNR.

	Reconstructed images from isotropic sequence	Directly acquired non-isotropic images	p	t
Sagittal Plane	26.12 ± 7.49	22.83 ± 7.10	0.001	-3.490
Coronal Plane	25.24 ± 7.61	22.28 ± 7.35	0.002	-3.225

### Relative tumor contrast

The relative tumor contrast between tumor to myometrium was not statistically different for the directly acquired vs. reconstructed either sagittal or coronal images (Table 4).

**Table 4:** Relative tumor contrast between tumor to myometrium.

	Reconstructed images from isotropic sequence	Directly acquired non-isotropic images	p	t
Sagittal Plane	0.16 ± 0.09	0.16 ± 0.10	0.642	-0.466
Coronal Plane	0.18 ± 0.10	0.20 ± 0.12	0.171	1.382

**Fig 3:**
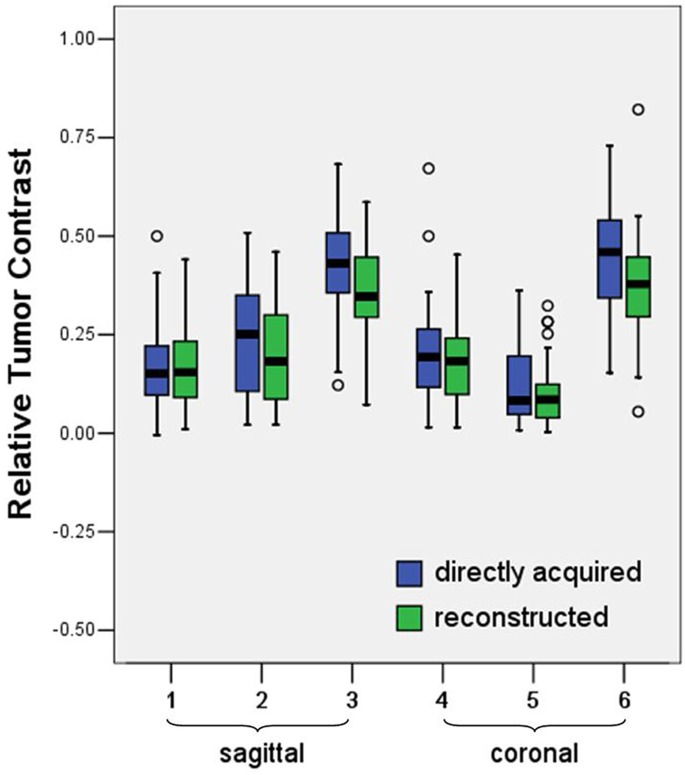
Box and whisker plots of relative tumor contrast. The relative tumor contrast between tumor to gluteal muscle and between tumor to fat in the directly acquired were higher than those in the reconstructed images (*p* = 0.001 and 0.031, sagittal; both *p* < 0.001, coronal). While the relative tumor contrast between tumor to myometrium was not statistically different for directly acquired and reconstructed either on sagittal or coronal images (*p* = 0.642 and 0.171, respectively).

The relative tumor contrast between tumor to gluteal muscle and tumor to fat were all lower in sagittal and coronal plane reconstructed images compared those in the conventional directly acquired non-isotropic images (Table 5 and 6, Fig. 3).

**Table 5:** Relative tumor contrast between tumor to gluteal muscle.

	Reconstructed images from isotropic sequence	Directly acquired non-isotropic images	p	t
Sagittal Plane	0.19 ± 0.11	0.24 ± 0.14	0.001	3.566
Coronal Plane	0.36 ± 0.12	0.43 ± 0.12	<0.001	7.199

**Table 6:** Relative tumor contrast between tumor to fat.

	Reconstructed images from isotropic sequence	Directly acquired non-isotropic images	p	t
Sagittal Plane	0.09 ± 0.03	0.12 ± 0.05	0.031	2.208
Coronal Plane	0.37 ± 0.12	0.45 ± 0.13	<0.001	4.523

### Local staging

**Fig 4:**
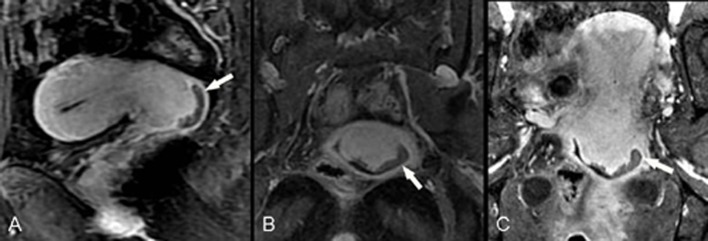
F55, poorly differentiated squamous cell carcinoma (stage IIA1). Directly acquired contrast-enhanced sagittal image (A) shows a tumor in the posterior cervical lip invading the vaginal fornix (arrow), with relative lower signal compared with the surrounding cervical stroma. Directly acquired contrast-enhanced coronal image (B) fails to show the entire uterus and the relationship between the lesion and vagina due to uterine anteversion. CPR from isotropic sequence (C) reconstructs the uterine and vaginal lesions in the same slice so that the relationship between the lesion and the vagina is more clearly displayed (arrow).

**Fig 5:**
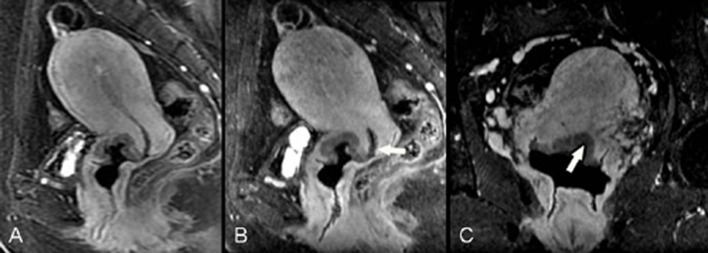
F47, poorly differentiated squamous cell carcinoma of the cervix (stage II A2) invades the vaginal fornix and cervical canal mucosa. Directly acquired contrast-enhanced sagittal image (A) shows a tumor in the anterior cervical lip with relative lower signal compared with the surrounding cervical stroma. Oblique sagittal image (B) and coronal CPR image (C), which were both reconstructed by the Isotropy, show more clearly that the lesion has spread to the cervical canal and vaginal fornix (arrow).

**Fig 6:**
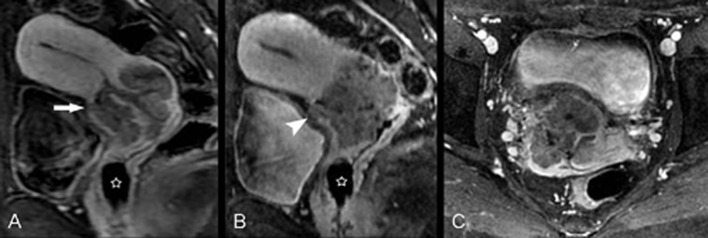
F28, poorly differentiated squamous cell carcinoma (stage II B) with negative cystoscopic findings. Pathology of intraoperative biopsy found cancer cells between the anterior wall of the cervix and bladder. Directly acquired contrast-enhanced sagittal image (A) shows a large cervical cancer lesion with relative lower signal compared with the surrounding cervical stroma. The anterior cervical stroma is suspicious interrupted (arrow). Sagittal image reconstructed by isotropic sequence (B) and axial isotropic image (C) clearly demonstrate that the anterior cervical stroma is interrupted by tumor (arrowhead). An OB tampons was placed inside the vagina (star).

Both reviewers showed higher accuracy when performing diagnosis of vaginal invasion by using the Isotropy (*p* = 0.039, reader1; *p* = 0.003, reader2) (Table 7, Fig. 4 and 5). No statistical difference was found when performing diagnosis of parametrial invasion using Isotropic vs. Three-planes (Fig. 6).

**Table 7:** Local tumor staging using the directly acquired sequence vs. 3D isotropic sequence.

Invasion	Sequences	Sensitivity	Specificity	PPV	NPV	Accuracy	p value^a^
Vaginal invasion	**Reader 1**							
	Three-planes	70%(7/10)	74%(45/61)	30%(7/23)	94%(45/48)	73%(52/71)	0.039
	Isotropy	70%(7/10)	85%(52/61)	44%(7/16)	95%(52/55)	83%(59/71)	
Parametrial invasion	Three-planes	100%(3/3)	97%(66/68)	60%(3/5)	100%(66/66)	97%(69/71)	1.000
	Isotropy	100%(3/3)	99%(67/68)	75%(3/4)	100%(67/67)	99%(70/71)	
Vaginal invasion	**Reader 2**						
	Three-planes	60%(6/10)	64%(39/61)	21%(6/28)	91%(39/43)	63%(45/71)	0.003
	Isotropy	60%(6/10)	82%(50/61)	35%(6/17)	93%(50/54)	79%(56/71)	
Parametrial invasion	Three-planes	67%(2/3)	97%(66/68)	50%(2/4)	99%(66/67)	96%(68/71)	1.000
	Isotropy	67%(2/3)	97%(66/68)	50%(2/4)	99%(66/67)	96%(68/71)	

^a^ *P* value was for comparison of accuracy between using the Isotropy and Three-planes.

When using the same sequences, both reviewers showed consistent performance in assessing vaginal and parametrial invasion (*k* value = 0.544–0.735; Table 8).

**Table 8:** Inter-reviewer consistency (*k* value).

	Three-planes	Isotropy
Vaginal invasion	0.544	0.724
Parametrial invasion	0.644	0.735

## Discussion

In recent years, both unenhanced and contrast-enhanced MR 3D scans have become increasingly popular in clinical practice. Compared with 2D scan, 3D scan is faster in speed, thinner in slice thickness without slice interval, higher spatial resolution and improved SNR, which is superior to (or at least equal to) 2D scan in distinguishing between lesions and normal structures at some anatomical locations [9,18–21]. Although the scan time is relatively longer when obtaining 3D isotropic high-resolution scan, reconstructed images has shown very high quality and demonstrated potentials to replace multiple planes of 2D scan for diagnosis [22].

Currently, studies on the 3D MRI of female pelvic lesions focused on the T2WI [18,23,24]. The studies [23] on the 1-mm-thick isotropic 3D T2WI sequence showed better or similar image quality and application value on detecting pathologic abnormalities, judging cervical cancer staging, and combined 3D T2WI and DWI sequences had superior or equal value for the endometrial carcinoma staging when compared to combined 2D T2WI and DCE-MRI [24]. Although T2WI can provide excellent unenhanced MR images of the uterus, the enhanced scan improves the contrast between cervical cancer and normal uterine tissues, and is especially important for discerning lesions with diameters less than 2 cm and post-treatment follow-up [14]. Currently, the contrast-enhanced scan of cervical cancer was usually performed in two or more directions by 2D or non-isotropic 3D sequence, which is with the expectation that more information regarding to relationship between lesions and surrounding structures can be revealed [15,25].

In this study, we compare the image quality of the latter three sequences after sagittal DCE sequence. Since these later three sequences are scanned at steady state time period and scanning time is relatively faster than the period of steady state, we assume that the enhancement difference for these three sequences can be omitted.

Our results showed that the Isotropy provided equal image quality and higher eSNR, compared to the non-isotropic sequence. The relative tumor contrast of tumor to myometrium, was similar in the reconstructed images vs. the non-isotropic sequence. However, both relative tumor contrast of tumor to fat and tumor to gluteal muscle were lower in sagittal and coronal reconstructed images compared with those in the conventional non-isotropic images, suggesting that compared to the reconstructed images, the conventional non-isotropic images probably had more accuracy when evaluating parametrial fat space and pelvic wall invasion. Nevertheless, this study showed that both reviewers’ diagnosis of parametrial invasion, based on the Isotropy, was similar to their diagnosis based on the Three-planes combined 3-mm-thick axial reconstructed image and conventional non-isotropic coronal and sagittal images. Possible explanation may lie in the fact that the Isotropy, based on 1-mm-thick axial data acquisitions has higher resolution than the other sequences in the axial direction. Therefore, it is better able to demonstrate smaller lesions and minute invasion, resulted in the advantage of determining parametrial invasion. In addition, their diagnosis of vaginal invasion of tumor, based on the Isotropy, was superiority to their diagnosis based on the Three-planes with single-direction respectively. The reason was that vaginal invasion from cervical cancer in this study was primarily invasion of the vaginal fornix which is a prominent curved structure, and the images of Isotropy with MPR and CPR could observe the relationship between tumor and the vaginal fornix in any direction, which further clarified whether the vaginal lesions were due to direct tumor extending or secondary to tumor protruding and compression, suggesting high spatial resolution of the Isotropy.

Furthermore, due to higher contrast between tumor and surrounding structures in the directly acquired images, it is important to select an appropriate scanning direction for 3D isotropic sequence in order to obtain the highest relative tumor contrast in that direction. For cervical cancer, radiotherapy and chemotherapy are often used instead of surgery, if the extent of tumor is beyond the parametrial tissue or one-third lower than the vagina. Physical examination and endoscopy can accurately determine tumor invasion in the lower one-third of the vagina, but they cannot assess the extent of pelvic invasion. Therefore, using MRI to assess whether tumor invasion is beyond the parametrial tissue will likely alter the clinical decision-making process. We chose the axial plane to perform 3D isotropic sequence due to that it can show the integrity of the cervical stromal ring, parametrial, bladder and rectum invasion clearly and more comprehensively than coronal or sagittal planes. In addition, radiologists are accustomed to axial images so that the results are more conducive to diagnosis and tumor staging. Finally, axial scan can display the regional lymph nodes in the obturator foramen or around the iliac vessels easily.

Our study had several limitations. Firstly, the diagnosis of cervical cancer and extent of tumor invasion in our patients were confirmed by surgical pathology, therefore, the majority of our patients were in early stage with relative small tumor in size. Our results show that Isotropy sequence has higher spatial resolution and can discriminate between a lesion and the surrounding tissues in any direction. Therefore, its advantages with regards to tumor staging should also be well studied in patients with more advanced cervical cancer. Secondly, in order to ensure high spatial resolution and SNR, the scan time of the Isotropy is longer and cannot be used for DCE-MRI. Only venous phase or delayed phase contrast-enhanced images can be acquired. Some studies have shown that DCE-MRI is more useful to evaluate the bladder and rectal invasion of cervical cancer [26–28]. Our study did not conduct further comparisons, but we believe that performing 3D contrast-enhanced isotropic sequence in delayed phase after DCE-MRI or combining those two sequences above may be more helpful in improving the accuracy of tumor staging. Finally, our study did not compare the Isotropy with the conventional non-isotropic sequence in the axial direction. Because the Isotropy, itself, acquires signals in the axial direction, there is no doubt that it has a higher resolution and improved ability to display small lesions due to its thinner slice. Hence, this study omitted the axial 3-mm-thick direct acquired sequence and replaced it with the axial 3-mm-thick reconstructed images when comparing the assessment of parametrial and vaginal invasion.

In summary, compared to conventional non-isotropic sequences, although the reconstructed images may reduce some relative tumor contrast, the 3.0T MR contrast-enhanced isotropic 3D-GRE-T1WI had shown better estimated SNR, superior to, or equal image quality and better tumor staging capability. All these findings have made 3D isotropic sequence an important role in preoperative MR staging of cervical cancer and worth exploring.
